# Impact of Hydraulic Well Restoration on Native Bacterial Communities in Drinking Water Wells

**DOI:** 10.1264/jsme2.ME14035

**Published:** 2014-10-02

**Authors:** Clemens Karwautz, Tillmann Lueders

**Affiliations:** 1 Institute of Groundwater Ecology, Helmholtz Zentrum München—German Research Center for Environmental Health, Ingolstädter Landstraβe 1, 85764 Neuherberg, Germany

**Keywords:** biofilms, drinking water, high pressure jetting, groundwater, pyrosequencing

## Abstract

The microbial monitoring of drinking water production systems is essential to assure water quality and minimize possible risks. However, the comparative impact of microbes from the surrounding aquifer and of those established within drinking water wells on water parameters remains poorly understood. High pressure jetting is a routine method to impede well clogging by fine sediments and also biofilms. In the present study, bacterial communities were investigated in a drinking water production system before, during, and after hydraulic purging. Variations were observed in bacterial communities between different wells of the same production system before maintenance, despite them having practically identical water chemistries. This may have reflected the distinct usage practices of the different wells, and also local aquifer heterogeneity. Hydraulic jetting of one well preferentially purged a subset of the dominating taxa, including lineages related to *Diaphorobacter*, *Nitrospira*, *Sphingobium*, *Ralstonia*, *Alkanindiges*, *Janthinobacterium*, and *Pseudomonas* spp, suggesting their tendency for growth in well-associated biofilms. Lineages of potential drinking water concern (*i.e. Legionellaceae*, *Pseudomonadaceae*, and *Acinetobacter* spp.) reacted distinctly to hydraulic jetting. Bacterial diversity was markedly reduced in drinking water 2 weeks after the cleaning procedure. The results of the present study provide a better understanding of drinking water wells as a microbial habitat, as well as their role in the microbiology of drinking water systems.

The need to protect drinking water from microbiological risks has been recognized for many years and strict regulations regarding the monitoring and maintenance of public drinking water production systems are in place (39). Groundwater is a major source of drinking water, constituting between 70–100 percent of the drinking water mix, especially in some European countries (46). To extract drinking water, wells are drilled vertically into the aquifer and reach between 30 to 100 m in depth. Wells consist of a well screen and well casing surrounded by filter gravel to ensure structural stability and production efficiency (10). The microbiome of drinking water produced from groundwater is primarily influenced by the influx of microbes from the surrounding aquifer, as well as by biofilms established in the drinking water wells and distribution network itself. Bacterial communities in drinking water distribution networks and also the impacts of disinfection and filtration have been extensively studied (11, 14, 23, 31, 33, 37, 42, 47). Although classical cultivation-based approaches are a powerful tool to detect specific indicator taxa in potable water, they only allow for a very limited grasp of total microbial communities (39).

*Proteobacteria* are the most dominant bacterial phylum in drinking water habitats, comprising up to 90 percent of total communities, represented mostly by *Alpha*-, *Beta*-, and *Gammaproteobacteria* (31, 37, 47). Some typical genera such as *Aquabacterium*, *Sphingomonas*, and *Polaromonas* (25, 31, 40, 42) have also been associated with extremely oligotrophic conditions. Other characteristic lineages frequently reported for drinking water communities are within the phyla *Bacteroidetes*, *Actinobacteria*, and *Nitrospira* (11, 24, 31). However, some genera that harbor potential pathogens are also frequently observed, such as *Aeromonas*, *Mycobacterium*, and *Legionella* (39). *Legionella* spp., in particular, appears to be ubiquitous in ground and drinking water systems, even at low temperatures, but is not necessarily connected to a pathogenic risk (43).

Although extensive research has been performed on microbes in drinking water systems, information regarding the ecology of groundwater extraction wells as a microbial habitat is limited. The impact of well microbiota on water quality, especially of attached microbiota established in the well and its surrounding casing, has not yet been specifically addressed. Biofilms are of potential concern as a possible seed bank for the survival and dispersal of pathogens in drinking water systems (2). However, biofilm microbiota can also have antagonistic effects on potentially entering pathogens, producing biocins and antibiotics (9).

Several environmental factors are known to control biofilm formation *e.g.* stress responses, communal behavior, or colonization of more favorable habitats. Surfaces in contact with water, even at low organic carbon concentrations, are generally colonized by microbial cells (3, 44, 47). In groundwater, the development of biofilms appears to be controlled by nutrient and energy inputs because extracellular polymeric substance (EPS) production requires microbes to invest resources. While biofilms in pristine, natural sediments are assumed to be restricted to patchy, monolayer communities (19), drinking water networks can experience substantial biofilm growth and the related unwanted effects, *e.g.* corrosion, clogging, or pathogen survival (2, 9, 35). These biofilms are continuously seeded by incoming aquifer microbes, but also develop their own niches and specific hydrochemical environments (38). Biofilms generally provide a habitat for more resistant and resilient microbial communities that are less susceptible to environmental stress.

Hydraulic well restoration by high pressure jetting is a routine maintenance method in porous aquifers that can be used to maintain well productivity in drinking water production by dislodging inorganic and organic deposits in the well casing (7). This purging event offers a unique possibility to access the microbes established in the well vicinity and discriminate them against the base influx of microbes from the surrounding aquifer. To the best of our knowledge, high pressure jetting has never been followed from a microbial community perspective. We hypothesized that (i) before this treatment, microbial communities from closely related wells in the same aquifer may be similar and dominated by lineages typical for low nutrient groundwater. (ii) The taxa specifically dislodged during the maintenance procedure may be distinct well microbiota established in the well matrix, potentially as biofilms. Furthermore, (iii) high pressure jetting should have a beneficial (= negative) effect on the detectability of bacterial lineages of potential pathogen affiliation after well restoration. In the present study, these hypotheses were examined in an operative drinking water production system in southern Germany during a routine hydraulic well purging event. The application of 454 pyrotag sequencing of bacterial 16S rRNA gene amplicons (30) to suspended microbes allowed for an extensive level of detail on the microbiota in this oligotrophic habitat.

## Materials and Methods

### Sample collection

Samples were taken before, during, and after hydraulic well restoration at an operational drinking water production unit in Baldham, east of Munich, Germany. Three groundwater extraction wells located in close proximity to each other (~50 m distance) were sampled in the summer of 2010. The wells extended ~37 m below ground into the Munich gravel plain, a quaternary aquifer dominated by fluvioglacial gravel deposits. The groundwater table was ~18 m below the surface, with the aquifer extending 9 to 14 m down to an underlying impermeable tertiary clay layer. A topographical sketch of the production site and its surroundings can be found under http://www.wasserverband-baldham.de/sektionen/technik/grundstueck.gif. Hydraulic conductivity in the proximity of the wells was typically high, with mean flow velocities between 10^−2^ to 10^−4^ m s^−1^. The wells were situated on a line perpendicular to groundwater flow; therefore, any hydraulic connectivity between them was excluded. Well 2 was distinct from the others in that it extended through an ~10 m layer of more sandy gravel. While well 3 was permanently used as a drinking water supply (pumping rate ~16 L s^−1^), wells 1 and 2 were reserve capacities and only operational for ~2 h per month (pumping rate ~100 L s^−1^). Well 2 had developed signs of reduced hydraulic conductivity over several years.

Four days before the actual purging event, fresh drinking water was collected from each well via dedicated monitoring faucets, into previously sterilized 5 L glass bottles and immediately transferred to the lab. Samples taken during high pressure jetting were collected directly from the operative suction hose at the beginning of purging and then after 15 and 45 min, which was also the end of the procedure. Fresh local groundwater was used for the cleaning procedure, thereby preventing the introduction of allochthonous microbes. The high pressure pump (up to 420 bar) was combined with a submersible rotating jet forcing water out of several nozzles at a speed of up to 180 m s^−1^. Suspended solids purged during jetting were collected in sterile 1-L glass bottles, transported to the lab, and centrifuged in a Beckman Coulter (Brea, CA) JA-10 rotor at 5,000 rpm for 15 min to collect sediment particles and attached biomass. A final water sample was taken two weeks after the jetting procedure.

Water for the dissolved organic carbon (DOC) analysis and ion chromatography was filtered with pre-rinsed syringe driven 0.45 μm PVDF-membrane filter units (Merck Millipore, Darmstadt, Germany) into prepared glassware. DOC was analyzed on a TOC-V (Shimadzu, Kyoto, Japan), while ions were examined on an ion chromatograph, DX-100 (Dionex, Sunnyvale, CA) (4). As with routine microbiological drinking water monitoring, 100 mL of water was filtered onto Endo KS plates (Sartorius, Göttingen, Germany) to cultivate potential *E. coli* and coliform bacteria at 36°C for 24 h (8). As was also tested by a certified analytical laboratory 2 weeks after well restoration, all water quality-related microbial parameters were met (*i.e.* coliform bacteria were not detected).

### Molecular analyses

A total of 4 L of water was filtered via a 0.2-μm sterile filtration unit (Corning Inc., Corning, NY). Filters (cut with a sterile scalpel) or 0.2–0.4 g of sediment, respectively, were filled into bead-beating cups containing Zirconia beads (Roth, Karlsruhe, Germany) and nucleic acid extraction buffer. DNA was extracted with a combination of bead beating (FastPrep24, MP Biomedicals, Solon, USA) and chemical lysis using phenol-chloroform extraction (30).

The amplification of bacterial 16S rRNA gene fragments for pyrotag sequencing was performed as described previously (30). Briefly, 50 μL PCR reactions contained 5 μL 10 × Taq Buffer, 3 μL 25 mM MgCl_2_, 0.5 μL 20 μg μL^−1^ BSA, 0.5 μL 10 mM deoxynucleoside triphosphate, and 1 μL of template DNA. 0.3 μL 50 μM Primers, Ba27f (5′-aga gtt tga tcm tgg ctc ag-3′) and Ba519r (5′-tat tac cgc ggc kgc tg-3′) (22), which were extended as amplicon fusion primers with respective adapters, key sequence and multiplex identifiers (MID) as recommended by Roche (Penzberg, Germany). The amplification routine consisted of an initial denaturation step of 94°C for 5 min, 28 cycles of denaturation at 94°C for 30 s, annealing at 52°C for 30 s, extension at 70°C for 60 s, and a terminal extension at 72°C for 6 min. PCR products were visualized using standard agarose gel electrophoresis.

PCR products were purified with magnetic beads (AmPure kit, Beckman Coulter) following standard procedures. DNA concentrations were quantified fluorometrically using the Quant-iT PicoGreen dsDNA quantification kit (Life Technologies, Carlsbad, CA) on a Mx3000Pro qPCR system (Stratagene, La Jolla, USA) to titrate equimolar amounts of amplicons for multiplex sequencing on a 1/4 FLX plate region (10^9^ amplicons μL^−1^). Emulsion PCR (emPCR), the purification of DNA-enriched beads, and sequencing was performed following the manufacturer’s protocols with a 454 GS FLX pyrosequencer (Roche) using Titanium chemistry. Quality filtering of raw pyrosequencing reads was performed using the automatic amplicon pipeline of the GS Run Processor (Roche), with a slight modification concerning the valley filter (vfScanAllFlows false instead of TiOnly). The applied bidirectional pyrotag sequencing workflow has been described in detail previously (30).

### Data analysis

Initial sequence data processing was performed using mothur software (36). Trimmed sequences with <250 bp, more than 8 homopolymers, and/or more than one primer mismatch were discarded. Sequences were aligned to the SILVA-compatible alignment database. The mothur-implemented algorithms PyroNoise (minimum flow length = 360, and maximum flow length = 720) and Chimera.uchime were used to denoise sequences and remove amplification artefacts. The remaining sequences were binned into operational taxonomic units (OTUs) at a 97% sequence similarity cut-off using the average neighbor clustering algorithm. Phylogenetic trees were then constructed for overall library comparisons using Clearcut. The classification of sequences was accomplished using the Greengenes train set described by Werner *et al.* (41). Processed taxon abundances from both datasets for f- and r-reads were averaged to mean values per sample. All sequencing data has been deposited with the NCBI sequence read archive under the BioProject ID PRJNA245507.

Multivariate statistics were performed with a subset of the sequence data including all taxa contributing at least 1% (relative abundance) in one of the samples. All statistical analyses were conducted using R version 15.2.0 (32). The diversity index inverse Simpson (1/α) and expected OTU richness of rarefied samples were calculated using the vegan package (29). The Inverse Simpson concentration was recognized as the effective number of species (16), based on the mean frequency of species in an ecosystem. To test the robustness of our community analyses, we performed bootstrap resampling (*n*=1,000) followed by repeated diversity calculation. Rarefaction analysis was performed using the rarefy function of vegan to estimate rarefied species richness (Ŝ_n_) (29) based on the minimum number of sequences amongst all samples. Data was transformed using Hellinger distances and principal component analysis was computed using the prcomp function. The sequences and constructed phylogenetic trees of the forward and reverse reads were used to estimate β-diversity based on weighted Unifrac values (26).

## Results

### Water analyses

The drinking water produced at the site was of moderate mineralization, which was characteristic for the region (Ca^2+^ 76.1 mg L^−1^, Mg^2+^ 21.9 mg L^−1^, and HCO_3_^−^ 324.8 mg L^−1^). The between-well variability of hydrochemical parameters was minimal (Table 1), with uniform temperatures of 8.6°C and a pH of 7.4. The aquifer was a well-oxygenated, oligotrophic system (~0.5 mg L^−1^ DOC). SO_4_^2−^ (8.6 mg L^−1^) and NO_3_^−^ (13.9 mg L^−1^) were present as potential alternative electron acceptors for microbial respiration, whilst ammonium, nitrite, and phosphate were below detection limits. As regularly inspected by certified labs at the site, our standard screening for coliform indicator bacteria in the drinking water via plating was also without a positive result.

### Variability of bacteria in drinking water wells

**Fig. 1:**
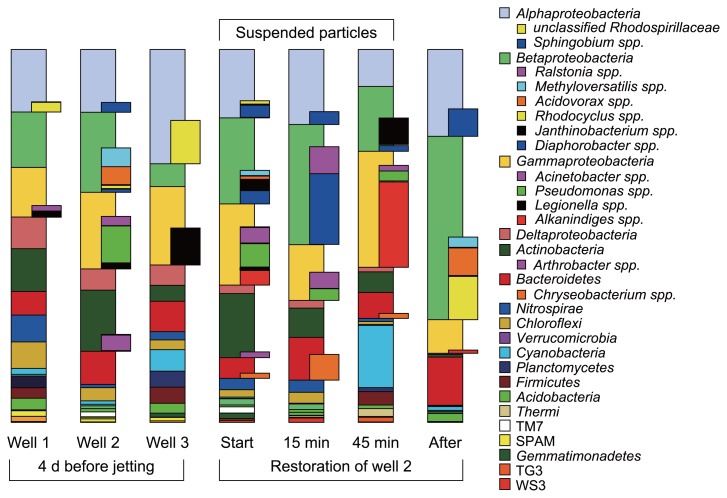
Relative read abundance of major taxa in bacterial pyrotag libraries of drinking water wells. Communities were analyzed between the three wells (planktonic bacteria), as well as in course of the hydraulic restoration of well 2 (suspended particle-associated bacteria) and after the event (planktonic bacteria). All phyla or classes contributing more than 1% abundance were depicted. The selected sub-phylum taxa described in the text are highlighted.

We attempted to assess inter-well variabilities in drinking water bacteria between parallel wells. After processing and quality filtering of all reads, sequencing provided 5,109 ± 933 reads per library and sample (Table 2), of which 99.9 ± 0.04% were assigned to the domain *Bacteria*. Overall, 12 out of the 47 known bacterial phyla contributed with more than 1% relative abundance to at least one of the libraries generated for the three wells. The phylum-level read abundances already indicated some variability between well communities (Fig. 1). The inverse Simpson diversity measure indicated the highest diversity within well 1 (1/λ = 49.9, Ŝ_n_ = 266), but lower values for well 2 (1/λ = 41, Ŝ_n_ = 245) and well 3 (1/λ = 27.1, Ŝ_n_ = 155). These results were further supported by the outcome of bootstrap resampling of community data (Table 2). At the same time, weighted UniFrac analysis suggested differences between overall community structures just below significance thresholds (W = 0.97, *p* = 0.06). *Proteobacteria* predominated the communities, contributing to ~half (57.2 ± 5.6%) of all reads within the wells before maintenance (Fig. 1). Within the *Proteobacteria*, *Alphaproteobacteria* (20.3 ± 6.5%), *Gammaproteobacteria* (17.3 ± 3.6%), and *Betaproteobacteria* (13.3 ± 5.9%) were the most abundant. A sub-phylum level perspective revealed the prevalence of members of the *Sphingomonadaceae* (5 ± 1%), *Comamonadaceae* (4.2 ± 2.9%), *Legionellaceae* (4.4 ± 3.9%), and *Pseudomonadaceae* (3.4 ± 4.6%) in all wells, with the latter two being of interest as ubiquitous lineages harboring potential drinking water pathogens. As indicated already by cultivation-based coliform screening, members of the *Enterobacteriaceae* and coliform bacteria were of extremely low abundance (<0.05%), or not detected at all. *Actinobacteria* only markedly contributed in well 1 (10.5%) and well 2 (15.5%). Well 3 displayed the lowest ratio of *Betaproteobacteria* (5.9%) and the highest ratio of *Alphaproteobacteria* (29.5%), dominated by *Rhodospirillaceae* (10.5%). Well 3 also hosted a high frequency of reads within the *Cyanobacteria* (4.1%). Sequences obtained from candidate phyla adding up to more than one percent in one of the communities were affiliated to the phyla *Thermi*, TM7, SPAM, TG3, and WS3 (Fig. 1).

### Bacterial dynamics during the restoration of well 2

**Fig. 2:**
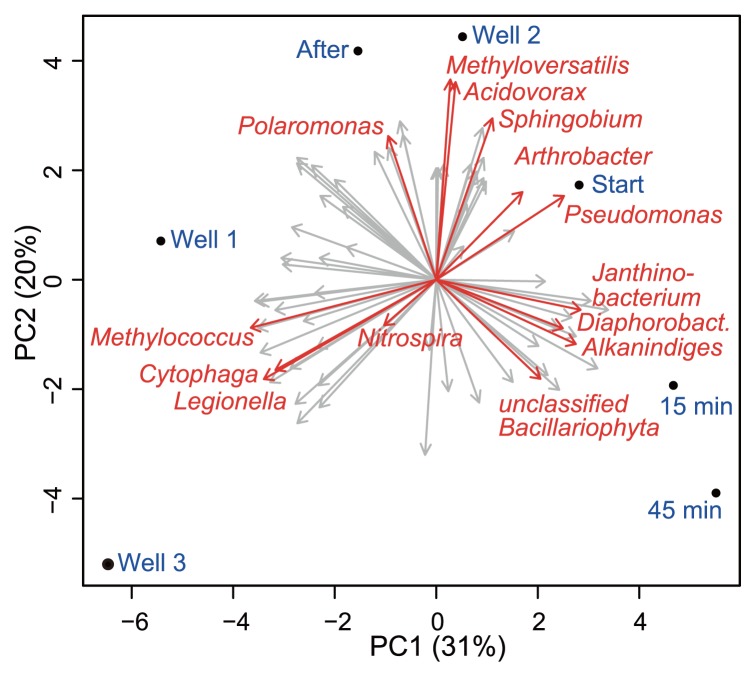
Principal component biplots of community variabilities between wells and during the maintenance of well 2. Sample codes are as in Fig. 1. Selected taxa with high impact on sample ordination are highlighted (arrows). Variance explanation ratios are given for each principal component (PC).

Four days after this initial assessment of well microbiota, we monitored bacterial community dynamics during the hydraulic purging treatment of well 2. The influence of high pressure jetting was clearly evident on the overall phylum-level community composition (Fig. 1). Only minor changes were observed in the community composition of suspended solids collected at the start of jetting from that of the initial water sample, as shown also by the principal component analysis of pyrotag data (Fig. 2). However, the abundance of *Betaproteobacteria* increased from ~22% to ~32% within 15 min of jetting, and members of the genus *Diaphorobacter* in particular appeared to have been preferentially purged. Although almost absent before and at the beginning of purging, reads of *Ralstonia* and *Chryseobacterium* spp. emerged in the sample after 15 min of jetting. None of these three taxa appeared in higher numbers in later samples. Moreover, the reads of *Acinetobacter*, *Nitrospira and Sphingobium* spp. were the most abundant in the purged samples collected at the first two time points. The reads of *Alkanindiges* spp. (22.9%) and also cyanobacterial sequences (7.7%) affiliated to *Bacillariophyta* as well as *Janithobacterium* spp. (7.1%) were observed after 45 min of maintenance (Fig. 1, 2).

Reads affiliated to *Pseudomonas* spp. constantly decreased in abundance during maintenance and were hardly detectable after 2 weeks. The water sample taken two weeks after high pressure jetting was of the lowest observed bacterial diversity (1/λ =15.9, Ŝ_n_ = 107), and more similar according PCA (Fig. 2) to well 2 before cleaning than samples towards the end of purging. The well community was dominated by *Betaproteobacteria* (48.8%), while *Actinobacteria* were almost absent (0.7%). The most abundant genus-level representatives were *Acidovorax* spp. (7.7%), *Sphingobium* spp. (7.4%), and *Rhodocyclus* spp. (11.7%) as well as unclassified sphingobacterial sequences (11.1%). The only taxon of potential pathogen affiliation that increased in abundance after the cleaning procedure was *Chryseobacterium* spp. (6.9%) within the *Bacteroidetes*.

## Discussion

### Well populations and variability

Bacterial communities in wells before and during purging were analyzed via bidirectional amplicon pyrotag sequencing. Although the reproducibility and semi-quantitative rigor of pyrosequencing libraries remains a matter of debate, we recently confirmed the strong reproducibility of taxon abundances across biologically replicated DNA extracts for our pyrotag workflow, and showed that relative abundances for taxa with a relative abundance between 0.2% and 20% can be semi-quantitatively meaningful (30). Therefore, mainly due to the resources available for this study, analyses of replicated water samples per well or time point were not performed here. The only two samples that could cautiously be considered as replicates, taken from well 2 before and upon the start of restoration, showed similar phylum-level taxon abundances. Nevertheless, we are aware of this central limitation of our sampling design, but are confident that our analyses still allow the most relevant community distinctions for the observed well microbiota to be discussed.

The diversity of drinking water bacterial communities has generally been reported to be high (23, 33, 47), and this was also confirmed in the present study. The prevalence of *Proteobacteria* was expected because the *Alpha*-, *Beta*-, and *Gamma*-subclasses have previously been identified as predominant taxa in potable water and drinking water biofilms (31, 37, 42, 47). Members of the *Actinobacteria*, *Bacteroidetes*, *Firmicutes*, and *Planctomycetes*, as well as *Cyanobacteria* are also frequent constituents of these communities (18, 33). Moreover, several unidentified candidate phyla (*e.g.* Thermi and TM7) were detected in our study, confirming their general presence in potable water samples (15, 23). The re-occurrence of these taxa in drinking water systems may reflect the specific conditions of groundwater habitats. The paradox of finding such high microbial diversities in oligotrophic systems could be related to the complexity of the habitat, the role of bacteriophages, or even the distinct dispersal mechanisms of community members.

Several taxa representing relatively defined metabolic capacities were observed, *e.g. Nitrospira* spp. (nitrification), *Diaphorobacter* spp. (nitrification, denitrification) (21), or *Methyloversatilis* spp. (methylotrophy). Ammonia-oxidizing bacteria and nitrite-oxidizing bacteria are regularly detected in potable water (2, 5, 27) and this has been attributed to disinfection with chloramine. However, this treatment has never been applied to the drinking water wells investigated here, and points towards an influence of distinct nitrogen sources. Typical methylotrophic and methanotrophic taxa (*e.g. Methyloversatilis* spp. and *Methylococcus* spp.) were present at low, but still sizable read frequencies (up to 5%).

Despite the high hydraulic conductivity of the local aquifer, bacterial communities between wells differed in their diversity and structure. This may have been related to the different usage routines and production intensities of the wells as well as differences in the sediment composition, even though their water chemistries were very similar. While well 3 was in constant use, well 1 and well 2 were typically inactive over several weeks and then flushed intensively to inhibit clogging. A constant pumping flow may have enhanced the growth of more compact biofilms while communities that developed under a natural groundwater flow may have been more easily detached (1). Microbes in unused wells may be influenced more by the well environment than those in active wells, which experience a higher influx from the surrounding aquifer.

Well clogging and its accompanying reduction in hydraulic conductivity have been attributed to the production of low solubility gases, the precipitation and deposition of metals and CaCO_3_, and filtration of suspended particles (34). Microorganisms and especially biofilms play crucial roles in most of these processes. Microbial biofilms and the production of EPS change the physicochemical properties of their local environment. Microbes in bulk water were previously reported to be more susceptible to the depletion of nutrients than biofilm constituents (3). Taxa associated with strong EPS production such as *Arthrobacter* spp., *Cytophaga* spp., and *Rhizobium* spp., have been linked to bioclogging (34). A marked number (4.3%) of *Arthrobacter* spp. reads was found in well 2, as well as those of *Cytophaga* spp. in the other two wells (2% and 3.4%, respectively). The influence of biofilms in proximity to the wells on overall community structures in the drinking water produced can only be speculated upon. The differences observed in community compositions between wells suggest that the sampled bulk water biota could consist of a mixture of ‘background’ aquifer microbes and dispersed well-specific populations. Some well-specific taxa could have been identified by their high variability in relative abundance between wells, as observed for *Pseudomonas* spp., unclassified *Rhodospirillaceae*, *Legionella* spp., *Methyloversatilis* spp. and *Acidovorax* spp. Taxa present at similar abundances in all wells may be distributed by groundwater flow and represent common aquifer taxa displaying a low impact in principal component analysis (Fig. 2).

### Hydraulic well restoration

The ratio of well-specific potential biofilm bacteria in the effluent was expected to increase during physical removal via high pressure jetting. In our time series, several taxa were detected at transiently increased abundances, suggesting their establishment in the well vicinity. Strong fluctuations in taxa between different sampling time points suggest the high heterogeneity of communities in the well itself. *Diaphorobacter*, *Nitrospira*, *Sphingobium*, and *Ralstonia* spp. appeared to have been prevalently removed after the first 15 min. As they were less dominant at later time points (Fig. 1), we speculated that these populations were situated directly at the well–aquifer interface. At the third time point (45 min) of jetting, the transient dominance of *Alkanindiges* populations was accompanied by *Janthinobacterium* spp. (Fig. 1), a typical soil bacterium known to form biofilms. *Janthinobacterium* spp. and *Ralstonia* spp. have both been identified in drinking water systems (37, 40). Both taxa are well-known soil-borne bacteria likely belonging to a constant seeding community enriched in the well habitat.

Although *Cyanobacteria* have also been repeatedly found in drinking water systems (5, 18, 42), the appearance of cyanobacterial DNA at the end of the maintenance process was unexpected because there was no relevant surface water body (lakes or streams) within ~10 km of the upstream aquifer. After the sampling of drinking water with a direct surface water influence, Revetta *et al.* argued that *Cyanobacteria* could also survive in dark subsurface waters based on storage compounds (33). Unicellular, N_2_-fixing cyanobacteria lacking all genes for photosynthesis have also been described (45) and several *Bacillariophyta* have been recognized as characteristic soil microorganisms (28). Hydraulic events such as rainfall and snowmelts have been shown to mobilize top soil bacteria into the subsurface with seepage water (6). In another recent study (15), high numbers of *Cyanobacteria* were detected in chlorinated drinking water stemming directly from an aquifer. Even the clogging of groundwater sediments has been attributed to *Cyanobacteria*, connected to EPS secretion, and related to calcium carbonate precipitation (20). Taken together, these findings suggest that *Cyanobacteria* may be able to survive and spread in the shallow subsurface, in spite of the adverse conditions to their usually phototrophic lifestyle.

The cleaning procedure markedly reduced bacterial diversity in the water of well 2. We speculate that high pressure jetting actually reduced the diversity of microbial niches in the vicinity of the well previously established by microbial colonization, filtration, and precipitation processes. The relative abundance of *Actinobacteria*–related reads decreased in each successive sample and was almost absent after two weeks. The dominance of *Betaproteobacteria* two weeks after cleaning could be a further indication for the reductions in biofilm bacteria, often belonging to the *Alpha*-, *Gamma*-, and *Deltaproteobacteria* (11, 24). In contrast, the specific taxa that were more abundant 2 weeks after cleaning appeared to represent the more mobile fraction of the aquifer microbes, and included ‘typical’ drinking water representatives such as *Rhodocyclus*, *Sphingobium*, or *Polaromonas* spp. (25, 40, 42).

Lineages harboring potential pathogens of drinking water (*i.e. Legionellaceae*, *Pseudomonadaceae*, and *Acinetobacter* spp.) reacted distinctly to hydraulic jetting. As described above, the read abundance of *Pseudomonas* spp. decreased steadily during well restoration, and was almost absent after 2 weeks. This result suggested that they were contributing to the attached microbiota in the well vicinity rather than in the aquifer itself. Therefore, a positive effect of hydraulic jetting on microbiological drinking water quality can be inferred. However, given the ubiquity and versatilities of *Pseudomonas* spp. and *Acinetobacter* spp. in aquatic environments, the impact of this purging on hygienic parameters may be hard to ascertain. Furthermore, the appearance of reads related to *Chryseobacterium* spp. (40) during cleaning indicates its presence in the well vicinity. In contrast, the reads of *Legionellaceae* were identified in all samples, but at decreased abundance during the actual purging event. This results emphasized the omnipresence of these bacteria in oligotrophic drinking water systems (43), but contradicted their establishment in biofilms in the vicinity of the well.

From an ecological perspective, the cleaning procedure has to be seen as a disturbance to the well ecosystem. The dynamic equilibrium model (13) predicts that, in low productive environments in which species have slow growth rates, infrequent disturbances are sufficient to promote invasion, which changes community compositions. Phylogenetically more diverse communities are less susceptible to invasion, and this can be linked to more efficient resource use within more diverse communities (17). The well microbiome presents a seed bank dispersing cells into the drinking water supply system, potentially all the way to the tap. The recolonization of such heterogeneous and oligotrophic habitats is difficult to predict after well restoration. While ‘niche-assembled communities’ could predict the co-existence of species because of microbial niche differentiation, ‘dispersal-assembled communities’ are determined by microbial dispersal, attachment, and persistence, independent of co-existing microbes (12). Based on the singular field observation reported here, it is impossible to conclude on the impacts of well restoration in a more general manner. The effects of well maintenance may vary in drinking water production systems with different hydraulic and geological settings. However, it is likely that the scheme of finding particular taxa that are more prone to disturbance, while others preferentially persist or recolonize the system relates to very general ecological traits of the detected microbes, and could be especially apparent in such oligotrophic groundwater habitats.

## Conclusions

This is the first study dedicated to elucidating the diversity and community structure of bacteria in drinking water wells upon hydraulic well restoration. Communities were distinct between different wells of the same production system, and this may have been due to the different usage practices of the wells and local aquifer heterogeneity. The pyrotag libraries generated here were capable of inferring relative community compositions in a robust manner (30). However, they did not provide quantitative data that could be attained via quantitative PCR or cytometric or microscopic cell counting. The quantitative impacts of well restoration and expected reductions in specific microbial populations in produced drinking water should be monitored in the future. In the present study, well restoration reflected highly dynamic community changes. The dominance of a few taxa at discrete time points indicated the preferential removal of populations from the well habitat. This allowed an insight into the attached bacteria in the actual well vicinity, and the ecology of their community assembly. Thus, the results of the present study contributed to a more integrated understanding of drinking water production systems from a microbial ecology perspective.

## Figures and Tables

**Table 1:** Hydrochemical parameters of drinking water produced from wells 1–3 four days before the well restoration.

Concentration [mg L^−1^]	Ca^2+^	Cl^−^	Mg^2+^	Na^+^	NO_3_^−^	SO_4_^2−^	DOC
Well 1	76.4	7.4	21.9	2.6	13.9	8.6	0.53
Well 2	76.2	7.5	21.9	2.7	14	8.6	0.56
Well 3	75.8	7.4	21.8	2.6	13.8	8.5	0.41

**Table 2:** Number of trimmed and processed 454 sequencing reads of bacterial 16S rDNA gene pyrotag libraries from wells 1–3. Diversity and richness indicators were inferred as stated.

	4 days before			Restoration of well 2			
		
	Well 1	Well 2	Well 3	Start	15 min	45 min	After
Trimmed reads (f- & r-; >250 bp)	5406	6366	4718	5714	3695	7086	6687
Denoised reads (f- & r-; >250 bp)	4703	5746	4447	5269	3536	6085	5980
Inverse Simpson index (1/λ)	49.9	41	27.1	58.5	17.2	14.4	15.9
Resampled inverse Simpson index (1/λ_bootstrap_)	46.6	39.1	26.1	54.3	17	14.2	15.9
Rarefied species index (Ŝ_n_)	266	245	155	241	119	252	107
Total species richness	392	389	232	372	165	447	189
